# Predictors of Central Compartment Involvement in Patients with Positive Lateral Cervical Lymph Nodes According to Clinical and/or Ultrasound Evaluation

**DOI:** 10.3390/jcm10153407

**Published:** 2021-07-30

**Authors:** Giuseppa Graceffa, Giuseppina Orlando, Gianfranco Cocorullo, Sergio Mazzola, Irene Vitale, Maria Pia Proclamà, Calogera Amato, Federica Saputo, Enza Maria Rollo, Alessandro Corigliano, Giuseppina Melfa, Calogero Cipolla, Gregorio Scerrino

**Affiliations:** 1 Unit of Oncological Surgery, Department of Surgical Oncological and Oral Sciences, University of Palermo, Via del Vespro, 129, 90127 Palermo, Italy; giuseppa.graceffa@unipa.it (G.G.); federica.saputo92@gmail.com (F.S.); enzamaria13@gmail.com (E.M.R.); calogero.cipolla@unipa.it (C.C.); 2 Unit of General and Emergency Surgery, Department of Surgical Oncological and Oral Sciences, Policlinico P. Giaccone, University of Palermo, Via L Giuffré, 5, 90127 Palermo, Italy; gianfranco.cocorullo@unipa.it (G.C.); irenevitale93@gmail.com (I.V.); mariapiaproclama@gmail.com (M.P.P.); calogera.amato92@gmail.com (C.A.); irene_melfa@yahoo.it (G.M.); 3 Unit of Clinical Epidemiology & Tumor Registry, Department of Laboratory Diagnostics, Policlinico P. Giaccone, University of Palermo, Via L Giuffré, 5, 90127 Palermo, Italy; mazzolasergio@hotmail.it; 4 Unit of Endocrine Surgery, Department of Surgical Oncological and Oral Sciences, Policlinico P. Giaccone, University of Palermo, Via L Giuffré, 5, 90127 Palermo, Italy; alessandro-corigliano@hotmail.it (A.C.); gregorio.scerrino@tiscali.it (G.S.)

**Keywords:** papillary thyroid carcinoma, central compartment, lateralcervical lymph nodes, EU-TIRADS, Bethesda, central neck dissection, lateral neck dissection, skip metastasis

## Abstract

Lymph node neck metastases are frequent in papillary thyroid carcinoma (PTC). Current guidelines state, on a weak level of evidence, that level VI dissection is mandatory in the presence of latero-cervical metastases. The aim of our study is to evaluate predictive factors for the absence of level VI involvement despite the presence of metastases to the lateral cervical stations in PTC. Eighty-eight patients operated for PTC with level II–V metastases were retrospectively enrolled in the study. Demographics, thyroid function, autoimmunity, nodule size and site, cancer variant, multifocality, Bethesda and EU-TIRADS, number of central and lateral lymph nodes removed, number of positive lymph nodes and outcome were recorded. At univariate analysis, PTC location and number of positive lateral lymph nodes were risk criteria for failure to cure. ROC curves demonstrated the association of the number of positive lateral lymph nodes and failure to cure. On multivariate analysis, the protective factors were PTC located in lobe center and number of positive lateral lymph nodes < 4. Kaplan–Meier curves confirmed the absence of central lymph nodes as a positive prognostic factor. In the selected cases, Central Neck Dissection (CND) could be avoided even in the presence of positive Lateralcervical Lymph Nodes (LLN+).

## 1. Introduction

Papillary thyroid carcinoma (PTC) has an extremely strong tendency to metastasize to the neck lymph nodes. This condition can occur in up to 80% of cases [1,2]. There is widespread acceptance of the classification of neck lymph nodes into seven levels, with level VI being the lymph nodes of the central compartment and level VII the lymph nodes of the upper mediastinum. Level I (sub-mental and sub-mandibular lymph nodes) is generally not considered in PTC exeresis, whereas levels II to V are the lymph nodes of the lateral compartment involved in neck dissection [3].

The current ATA guidelines (2015) regarding prophylactic central neck dissection (PCND) limit this indication to stages from T3 (tumor > 4 cm in greatest diameter and/or gross invasion of prethyroid muscles), while therapeutic central neck dissection is indicated in T1 (tumor less than 2 cm) or T2 (tumor size between 2 and 4 cm) only in the clear presence (clinically, ecographically and/or biopsy proven) of central metastases, or in cases of lateral lymphadenopathy [4,5,6]. Although widely accepted and practiced, this indication, contained in recommendation 36 of the ATA guidelines, is weak, with a low level of evidence [4].

Central neck dissection is not a procedure without complications. Hypoparathyroidism and recurrent laryngeal nerve palsy (both transient or permanent) may occur more frequently than in simple thyroidectomy [7,8,9,10,11,12].

The realization that central neck dissection is a surgical procedure with additional morbidity may lead to the search for a context in which, even in the presence of metastases to the lateral neck lymph nodes, central neck dissection could be avoided. The occurrence of skip metastasis to the lateral lymph node compartment is well known [13,14,15]. However, only recently a study appeared in the literature specifically aimed at answering the question of whether prophylactic central compartment dissection is always needed in the presence of lateral neck metastases [16].

The aim of this study is to evaluate predictive factors for the absence of level VI involvement despite the presence of metastases to the lateral cervical stations in PTC, and also to formulate hypotheses on the possible risk of persistence or recurrence directly related to the persistence of level VI lymph nodes.

## 2. Material and Methods

This retrospective observational cohort study was carried out on patients consecutively that had undergone thyroidectomy from January 2010 to December 2020, with the diagnosis of papillary thyroid carcinoma at two University Surgery Units: the General and Emergency Surgery Unit and the General and Oncology Surgery, both of which are referral centers for endocrine neck surgery in western Sicily. All patients with complete clinical reports regarding preparation for surgery, hospital course and follow-up until 30 June 2021 were included in the study; patients included underwent surgery for PTCs with metastases to the lateral-cervical lymph nodes at the time of surgery and who, therefore, underwent TT + CND + unilateral LND in one step. Surgical procedures were performed by surgeons, belonging to the respective operating units, classifiable as “high volume” according to the unanimous consensus of the international literature [17,18], having all performed over 1000 thyroidectomies with more than 100 procedures/year for a period of activity of 10 or more years.

Exclusion criteria were: surgery for central and/or latero-cervical metastases, which, therefore, underwent Thyroidectomy and/or Central Neck Dissection and/or Lateral Neck Dissection in two or more steps. We also excluded non-papillary thyroid cancers, patients with incomplete clinical documentation, those with malignancy of another site or who had developed an extrathyroid cancer during follow-up, familial thyroid tumors and who had undergone operations performed by operators with a volume of activity of less than 1000 total thyroidectomies or <100 thyroidectomies/year or with a period of “dedicated” activity of less than 10 years.

We considered the following variables (in brackets, the way the variables were assessed): age (continuous), sex (categorical), TSH values detected at the time of preparation for surgery (continuous), autoimmunity (categorical), largest diameter of the nodule measured at ultrasound (continuous), cancer variant (classical, follicular, other, categorical). Based on preoperative ultrasonographic findings, we included the suspicious lesions in the excel sheet classifying them as located in the “upper lobe pole” (=1), “middle lobe” (=2) and “other” (=3), including in this group the isthmic or paraisthmic lesions and the lesions at the lower pole of the thyroid lobe (categorical variable). Moreover, we recorded multifocality (categorical), Bethesda classification (categorical), EU-TIRADS classification (categorical), total number of central lymph nodes removed (continuous), number of positive central lymph nodes (continuous), total number of lateral lymph nodes removed (continuous), number of positive lateral lymph nodes (continuous) and outcome (unfavorable, yes/no, categorical). The categories that we considered as “unfavorable” outcomes fell into two different patterns: patients with “persistence”, in whom there was proven locoregional disease before six months after postoperative radioiodine ablation, and patients with “recurrence”, in whom new disease occurred after this cut-off time. The presence of locoregional disease (persistence or recurrence) was assessed by integrating the results of laboratory tests (thyroglobulin [Tg] > 10 ng/mL after appropriate discontinuation of L-Tyroxine treatment in the absence of anti-Tg antibodies), ultrasound (presence of suspected locoregional tissue) and fine-needle aspiration biopsy of tissue detected on ultrasound, which in turn was evaluated by cytology and Tg assay on the eluate. In two cases, there was persistence of elevated Tg values in the absence of locoregional recurrence on instrumental exams: these patients were excluded from the study because of the uncertainty of considering them as loco-regional recurrence, systemic recurrence or false positive result.

For the purpose of the Kaplan–Meier curves, the date on which the recurrence was detected was reported. Disease-free patients were conventionally verified as of 30 June 2021.

Among the variables included in the statistical evaluation, location within the thyroid lobe was documented in the literature as a risk factor for skip metastasis [13,14,15], while variables such as the threshold value of metastatic lateral lymph nodes and the total number of central lymph nodes removed were derived from the study results. TIRADS and cytology (classified according to Bethesda) were included in order to evaluate whether these two simple datapoints from the preoperative diagnostic procedure could be used as indicators and guide the choice of surgery.

### 2.1. Surgery

The surgical procedure has always been performed as a ‘formally’ total thyroidectomy. In the patients undergoing Intraoperative Nerve Monitoring, if the surgical protocol required to suspend the operation at lobectomy with removal of the central hemicompartment ipsilateral to the cancer (two-staged thyroidectomy), we excluded these patients (two in the whole cohort) from the study. Central neck dissection was always bilateral, with excision of both periricurrential and paratracheal chains, and lateral neck dissection extended at least to IIa-III-IV levels, while IIb and V levels were removed in the presence of gross and/or multiple latero-cervical metastases detected by ultrasound or in the presence of involvement of these stations.

### 2.2. Radioiodine Ablation and Endocrinological Treatment

All patients with metastatic lateral lymph node (LLN+) underwent radioiodine ablation, at a dosage of about 100–150 mCi, after suspension of L-thyroxine treatment until TSH values > 30 microIU/mL were reached. This treatment was performed 4–8 weeks after surgery and, after post-treatment whole-body scan, suppressive therapy with L-thyroxine was reimposed with the aim of bringing TSH to about 0.5 microIU/mL.

### 2.3. Statistical Analysis

In a first step, a univariate analysis was carried out in which Fisher’s exact test for categorical variables and Mann–Whitney’s test for continuous variables were applied.

ROC curves were then realized in order to evaluate the accuracy of the variables “number of central lymph node (CLN)”, “number of lateral lymph node (LLN)” and “number of LLN+” as predictors of the occurrence of metastatic central lymph node (CLN+ ≥ 1).

Moreover, a multivariate logistic analysis was performed in which the following variables were included: Age, Sex, TIRADS, Location, CLN and LLN+. From the various models, the best fit was chosen. Akaike’s Information Criterion (AIC) was used as an indicator of the quality of the fit of the multiple logistic regression function to the needs of the study.

Finally, Kaplan–Meier curves were created in the following three groups: CLN+ = 0, All cases and CLN+ ≥ 1. The statistical significance of the differences between the Kaplan–Meier curves was checked with the Log Rank test.

Statistical elaborations were carried out with the software RStudio (version 3.4.1 of 30 June 2017) for R (version 2.1) The ROC Curves and Kaplan–Meier were made using the application packages “pROC” and “Survival”.

## 3. Results

Eighty-eight patients in follow-up from 6 months to 10 years (mean 4.6 years) with a mean age of 46 years (range: 14–82) met the inclusion criteria.

### 3.1. Univariate Analysis

**Figure 1:**
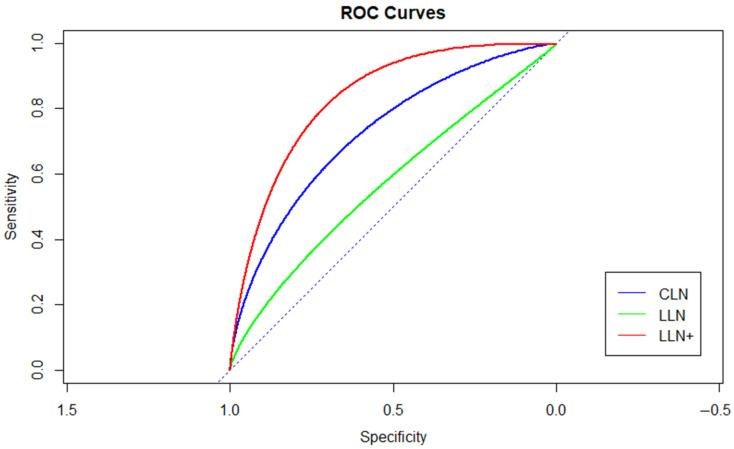
The number of LLN is a poor predictor of CLN+ ≥ 1.

From the univariate analysis (Table 1) the variables with *p*-value < 0.05 were: Location, TIRADS, CLN and LLN+. The location, TIRADS and LLN+ seem to be a moderately accurate predictor of CLN+. Comparing the ROC curves, in which the specificity/sensitivity relationship was calculated using CLN+ as a reference we note that the number of LLN is a poor predictor of CLN+ (Figure 1). In fact, the Area Under the Curve (AUC) values were = 0.57 and 95% confidence interval (CI) (0.44–0.68), with a threshold value of 28.5. Specificity, therefore, was 1% CI (1–1) and sensitivity = 0.18 with 95% CI (0.09–0.29). The numbers of CLN and LLN+ were found to be a moderately accurate predictor of CLN+ with AUC = 0.73 and 95% CI (0.61–0.82) with a cutoff value = 7.5, specificity = 0. 84% CI (0.72–0.97) and sensitivity = 0.46 with 95% CI (0.34–0.59) and AUC = 0.83 and 95% CI (0.73–0.92) with a cutoff value of 2.5, specificity = 0.81% CI (0.66–0.94) and sensitivity = 0.77 with 95% CI (0.66–0.88), respectively.

### 3.2. Multivariate Analysis

The multivariate analysis (Table 2) revealed that the best-fitting model according to AIC (=67.98) included the following variables: Age, Sex, Localization, CLN > 7 and LLN+ < 4. The model shows that center-lobar cancer localization and LLN+ < 4 appear to be protective values (CLN+ = 0 and no recurrence) with ORs of 0.005 95%IC (8.12 × 10^−5^–8.21 × 10^−2^) (*p*-value < 0.05) and 0.020 95%IC (7.81 × 10^−4^–0.164) (*p*-value < 0.05), respectively.

**Figure 2:**
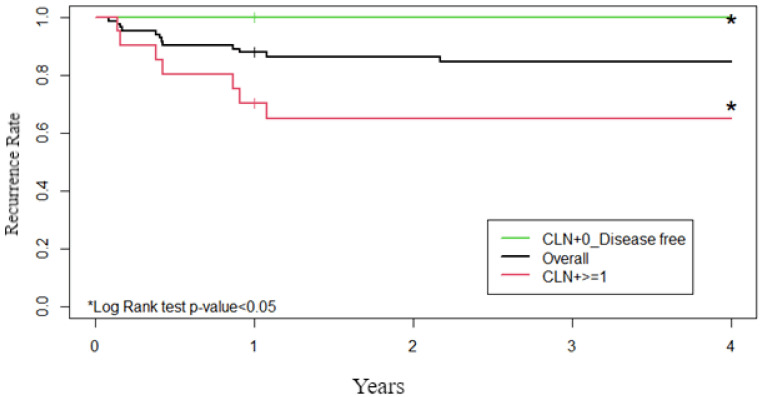
The group of patients with no central lymph nodes involvement has longer disease-free survival than the group with metastatic central lymph nodes (*p* < 0.05). Kaplan–Meier Curves.

From the analysis of Kaplan–Meier curves (Figure 2), it can be seen that the difference in disease-free survival between the group of patients in which no central lymph nodes were found and the group with metastatic central lymph nodes was significant (*p* < 0.05).

Finally, we found it interesting to note that patients with CLN = 0 had a small number of metastatic LLN (4 or less) and none of them experienced persistence or disease recurrence. 

Variables found to be non-significant in the univariate analysis (age, sex, TSH levels, autoimmunity, multifocality, Bethesda category, TIRADS category) were not considered in the multivariate analysis.

Variables found.

## 4. Discussion

Nowadays, the role of PCND in PTC is still debated, with studies proposing to avoid this procedure for early stages and limit CND to cases of clinically positive central lymph nodes [21,22] and others showing benefits even for stages below T3 [23,24,25]. Given the advantages, especially from the point of view of postoperative radiometabolic treatment, and possible complications such as hypoparathyroidism and recurrent nerve palsy, careful case selection and consensus in the context of the multidisciplinary team has been proposed by others [26]. In any case, the position that PCND does not change the overall survival rate of PTC seems to be prevalent [27,28,29].

Currently, the ATA, CCN and ESES guidelines consider the presence of lateral neck metastases as an indication for CND [4,6,30]. This is also supported by the order of the lymphatic diffusion pathways, which, however, may vary for portions of thyroid tissue located near the upper poles of the gland, as stated in several studies [13,14,31,32,33]. This was also confirmed in a detailed anatomical study [34].

Central lymph node metastases in PTC are extremely frequent (up to 80%), but most of them are microscopic, detected after careful histopathological evaluation and have no clinical significance [2,16,35]. On the other hand, in studies considering prophylactic lateral cervical dissection, the occurrence of metastases in levels II–V was well above 50% [36], although there is very little support for such a choice and not much data available on the subject. These data have to be compared with those related to the risk of PTC recurrence, which is very low in the most common variants of PTC and, in any case, ranging from 1% to 40% [16,37], and, in an even clearer perspective, in the 5-year disease-free survival, which reaches 98% [4]. This debate could be concluded by stating that, obviously within certain limits, there is no correlation between central or lateral lymph node metastases, rate of locoregional recurrence and in general the prognosis of PTC [4,16,21,38]. These limits may be constituted by the real numerical and especially volumetric consistency of the affected lymph nodes [16], as well as by the prognostic factors of cancer, which, however, most likely act independently from lymph node metastases [39].

The attitude taken at our institution with regard to PCND has gone from a gradual enthusiasm that had extended its indications also to early stages of PTC (T2 and sometimes T1), which culminated in the middle of the last decade [9,24], to a gradual alignment with the actual indications of the main scientific societies mentioned above, so that, in fact, we now perform PCND only in T3 and in N1b with any T.

In this study, we found a relapse rate of 13.7% (12), similar to percentage described in several other studies [40,41].

The number of patients with positive LLN but without central compartment involvement was very high (32 = 36.4%). This high prevalence of LLN+ without CLN+ would be sufficient to justify further investigation, which led to our results.

An aspect that we found interesting in our study was the finding that the presence of few positive LLNs (<4) seems to be protective compared to the presence of CLN metastases; therefore, in such circumstances, the presence of a sporadic metastasis in the lateral site might be predictive of skip metastasis. A finding that could be investigated in the future is the association of more advanced TIRADS with the increased likelihood of central metastases in the presence of known LLN metastases. Although we are not able to give a definite interpretation to this statistical finding, we believe we can explain this effect by a probably greater aggressiveness of the tumor that could tend to be associated with more advanced morphological aspects. A similar association was not found when evaluating cytological features with the Bethesda system, whose degree of alterations do not correlate in any way (indeed we did not expect it) with a more extensive metastatic spread of the tumor.

The first interesting result of our study is evidenced by the multivariate analysis, which confirms the importance of the apical location in the thyroid lobe as a risk factor for the presence of possible skip metastases and, conversely, a certain “protective” role of cancer locations other than this one in the context of the thyroid lobe.

Another attractive finding arising from the results of the Kaplan–Meier curves is the better disease-free survival reported in the group of patients without involvement of CLN, even with LLN+, demonstrating a role of the CLN− as a positive prognostic factor in terms of persistence or recurrence. Conversely, the simultaneous presence of CLN+ and LLN+ entails in a worse prognosis. All these results could constitute a further justification to limit prophylactic CND in the presence of isolated skip metastases, provided that these do not hide central metastases [40].

We are aware of several biases in our study: firstly, its retrospective nature; secondly, the expertise-dependence of lymph node clearing during lymph node dissections; and thirdly, we consider the study to be very large, with follow-up ranging from 6 months to 10 years. Furthermore, the empiricism with which recurrence was established and with which, in turn, it is distanced from persistence. Furthermore, the enrolment of latero-cervical dissections less extensive than II–V may constitute a bias but this type of surgical choice is justified and validated in the literature [41,42,43].

Another bias is the lack of evaluation of some prognostic factors, such as angioinvasiveness, extracapsular or extranodal extension, but usually, such evaluations are not reliably answered during the preoperative workup and, on the other hand, our intention was to find reliable answers to the question “what to do if a certain condition is found” in a real scenario. Finally, we did not compare a population of patients who underwent CLND *plus* LLND versus only LLND. In this perspective, the comparison between the two groups should be considered virtual.

## 5. Conclusions

At present, we cannot consider the data obtained from this study sufficient to lead to the adoption of new treatment protocols.

Far from providing definitive data on the existence of a category of PTC patients in whom level VI dissection can be avoided even in the presence of lateral cervical metastases, this study aims to provide further food for thought and add data to achieve the goal of avoiding central compartment dissection, albeit in a restricted category of patients, and thus limiting risks and sequelae of this not entirely harmless procedure.

## Figures and Tables

**Table 1:** The number of CLN and LLN+ were found to be a moderately accurate predictor of CLN+ ≥ 1. TIRADS Classification: TIRADS I: Normal thyroid US; TIRADS II: Benign Aspects; TIRADS III: Probably Benign Aspects; TIRADS IV A Low Suspicious Aspect; TIRADS IVB 1 or 2 signs of High suspicious aspects and no Adenopathy; TIRADS 5: ≥3 of High Suspicious aspects and/or Adenopathy. The Bethesda System for Reporting Thyroid Cytopathology: Bethesda I: Nondiagnostic or Unsatisfactory; Bethesda II: Benign; Bethesda III: Atypia of undetermined significance or follicular lesion of undetermined significance; Bethesda IV: Follicular neoplasm or suspicious for a follicular neoplasm; Bethesda V: Suspicious for malignancy; Bethesda VI: Malignant. Nodule location: Site 1: upper lobe pole; Site 2: middle lobe; Site 3: isthmic, paraisthmic or lower pole [19,20].

Variable	CLN+ 0_Recovery	CLN+ ≥ 1	Total	OR (95%CI)	*p*-Value
Age	47	46			0.677
Sex M	6	22	28	2.77 (0.92–9.60)	0.05849
Sex F	26	34	60		
Total	32	56	88		
TSH	2.19	2.15			0.5318
Autoimmunity YES	7	12	19	0.97 (0.31–3.32)	0.9999
Autoimmunity NO	25	44	69		
Total	32	56	88		
Size 1	27	44	71		0.8454
Size 2	2	6	8		
Size 3	3	6	9		
Total	32	56	88		
Site 1	12	31	43		**8.141 × 10^−6^**
Site 2	13	1	14		
Site 3	7	24	31		
Total	32	56	88		
Multifocality NO	40	9	49	0.37 (0.06–1.65)	0.2136
Multifocality YES	36	3	39		
Total	32	56	88		
Bethesda 1	1	1	2		0.3606
Bethesda 2	3	4	7		
Bethesda 3	9	11	20		
Bethesda 4	15	22	37		
Bethesda 5	4	13	17		
Bethesda 6	0	5	5		
Total	32	56	88		
TIRADS 3	2	1	3		**0.008372**
TIRADS 4	14	10	24		
TIRADS 5	16	45	61		
Total	32	56	88		
CLN	4.4	7.5			**0.0008545**
LLN	17.31	19.57			0.3917
LLN+	1.81	4.36			**5.741 × 10^−7^**

ROC Curves. Bold format of data mean *p*-Values < 0.05.

**Table 2:** The model shows that center-lobar cancer localization and LLN+ < 4 appear to be protective values.

Variable	OR	IC (Inf) 95%	IC (Sup) 95%	*p*-Value
Age	0.959	0.907	1.007	0.10725
Sex	0.564	0.073	3.448	0.54998
Site 2	0.005	8.12e^−5^	8.21e^−2^	0.00210 **
Site 3	1.224	0.275	5.491	0.78752
CLN > 7	1.587	1.231	2.195	0.00149 **
LLN+ < 4	0.020	7.81e^−4^	0.164	0.00238 **

** mean *p*-values lower than 0.05.

## Data Availability

Digital and paper archives of the Department of Surgical Oncological and Oral Sciences, Policlinico P. Giaccone, University of Palermo, Via L Giuffré, 5, 90127 Palermo, Italy.
